# Personal values in adolescence and their associations with metabolic biomarkers in adulthood: a Japanese population-based study

**DOI:** 10.1186/s13030-020-00197-5

**Published:** 2020-10-07

**Authors:** Natsu Sasaki, Kazuhiro Watanabe, Norito Kawakami

**Affiliations:** Department of Mental Health, Graduate School of Medicine, The University of Tokyo, 7-3-1, Hongo, Bunkyo-ku, Tokyo, 113-0033 Japan

**Keywords:** Adolescents, Metabolic syndrome, Public health, Body mass index, Epidemiology, Value of life

## Abstract

**Background:**

Personal values, which are formed in early life, can have an impact on health outcome later in life.

**Objective:**

The aim of this study is to investigate the relation between personal values in adolescence and bio-indicators related to metabolic syndrome (MetS) in adulthood.

**Participants and Methods:**

The longitudinal data used was from the Japanese Study on Stratification, Health, Income, and Neighborhood (J-SHINE). Personal values in adolescence were retrospectively obtained in 2017 from a self-reporting questionnaire, composed of value priorities and commitment to the values. Venous samples were collected in 2012 for low and high-density lipoprotein (LDL, HDL) cholesterol and hemoglobin A1c (HbA1c). Body mass index (BMI), waist circumference and systolic and diastolic blood pressure (SBP, DBP) were also measured. The associations of each variable were examined by partial correlation analysis. In addition, multiple linear regression analysis was conducted to examine overall associations between personal values and the sum of standardized scores (Z-score) of the biomarkers as a proxy of MetS.

**Results:**

The total population (*n* = 668) included 261 men and 407 women. For men, the personal value priority of “Having influence on society” was associated with high HDL cholesterol (0.133, *p* = 0.032) and “Cherishing familiar people” with low waist circumference (*r* = -0.129, *p* = 0.049), low SBP, and high DBP (*r* = -0.135, *p* = 0.039; *r* = 0.134, *p* = 0.041). For women, “Not bothering others” was associated with high SBP and low DBP (*r* = 0.125, *p* = 0.015; *r* = -0.123, *p* = 0.017). "Economically succeeding" was associated with a worse outcome (β = 0.162, *p* = 0.042) in men.

**Conclusions:**

Although some significant associations were found between personal values in adolescence and MetS-related markers in adulthood, the overall associations were not strong. Culturally prevailing values were likely to be associated with a good outcome of metabolic health.

## Introduction

Metabolic syndrome (MetS) recognizes risk factors of underlying cardiovascular and metabolic disease characterized by comorbidity of abdominal obesity, high blood glucose or insulin resistance, hypertension, dyslipidemia, and microalbuminuria [1]. At least one-fifth of the adult population is affected by MetS, with an increase in prevalence in Asia [2]. A recent meta-analysis showed that MetS was associated with a higher risk of all-cause mortality in the elderly [3]. Early prevention of MetS is of critical importance in Japan, a highly aged society. Obesity and overweight defined by body mass index and waist circumference are especially important components of MetS in developing cardiovascular disease and cancer and elevating the risk of mortality [3, 4]. Therefore, detecting and intervening in the modifiable risk factors for MetS in an early life stage are important issues in medicine and epidemiology [5].

Previous studies have indicated that individual psychological and behavioral factors are associated with the components of MetS [6–9]. MetS is usually considered a reflection of individual lifestyle, such as insufficient physical activity, excessive stress, and unhealthy eating habits [5]. For example, motivations for adherence to improving lifestyle and diet modification were key factors in the reduction of MetS components [6]. Moreover, these factors have been associated with biomarkers (e.g., serum cholesterol, triglyceride, glucose) related to Mets. As an example of the important role of psychological factors in physical health, stress management is known to be a direct mediator of the link between stress and preferable biomarkers related to MetS through emotional and behavioral self-regulation [8].

Children and adolescents have become important targets of public health because individual health-related psychology and behavior are formed in early childhood [10]. Previous review has indicated that early healthy practice can be protective for metabolic outcomes in adults [11, 12]. Psychosocial exposures in childhood, which influence the creation of a sense of value, can also be determinants of lifelong predilection to metabolic disease in adulthood [13–18]. There is an urgent need to explore the kind of psychological and behavioral factors in childhood and adolescence that affect the onset of MetS throughout the rest of the life course.

One such factor is personal value, which is developed in adolescence [19] and that can affect health, well-being, and the development of MetS in adulthood [20]. Personal values consist of value priorities [21] and commitment to values [22]. Personal value priorities, introduced by Schwartz [21], are a personal selection of important values. Value priorities are developed in adolescence and affect individual psychological and behavioral health, including personal choice of lifestyle and commitment to one’s own health [9, 20]. In addition, commitment to values, which indicates how committed people are to living their values, is considered important [22]. Behavioral commitment to values can be an intrinsic motivation and is known to have a positive relation with greater well-being [23]. Research on personal values in adolescence investigating the relation with outcomes in adulthood have recently begun to be published [24].

However, it is still unknown how much impact personal values in early life have on the risk of MetS later in life. Ascertaining whether or not there will be a direct association between personal values and health outcomes might be useful in considering the importance of values in adolescence. It seems worthwhile to investigate the relation between personal values in adolescence and bio-indicators related to MetS in adulthood. Elucidating this relation would have practical implications for MetS prevention. Although the focus is now mainly on the high-risk adult population, considering personal values in adolescence may shift the target to an earlier life stage. To reveal the specific association between personal values in adolescence and health status in adulthood would possibly contribute to the prevention of MetS in Japan, a highly aged society. This study investigates the association between personal values at age 15 with the biological markers related to MetS in adulthood. We explored and tested the interrelations between value priorities and commitment to those values, and low- and high-density lipoprotein cholesterol, HbA1c, body mass index, waist circumference, and blood pressure.

## Participants and Methods

### Study design

#### Study setting

This study was conducted by recall of personal values in adolescence, using longitudinal data from the Japanese Study on Stratification, Health, Income, and Neighborhood (J-SHINE), which has been described in detail elsewhere [25]. It was designed to investigate the complex associations between social factors and health. The ethics committee of the Graduate School of Medicine and Faculty of Medicine of The University of Tokyo approved this study. Consent for participation in the study and the agreement for publication was indicated by completing and returning the self-administered questionnaire. Additional written informed consent was obtained for participants who undertook physiological measurements and serum samplings.

#### Study period and participants

**Fig. 1:**
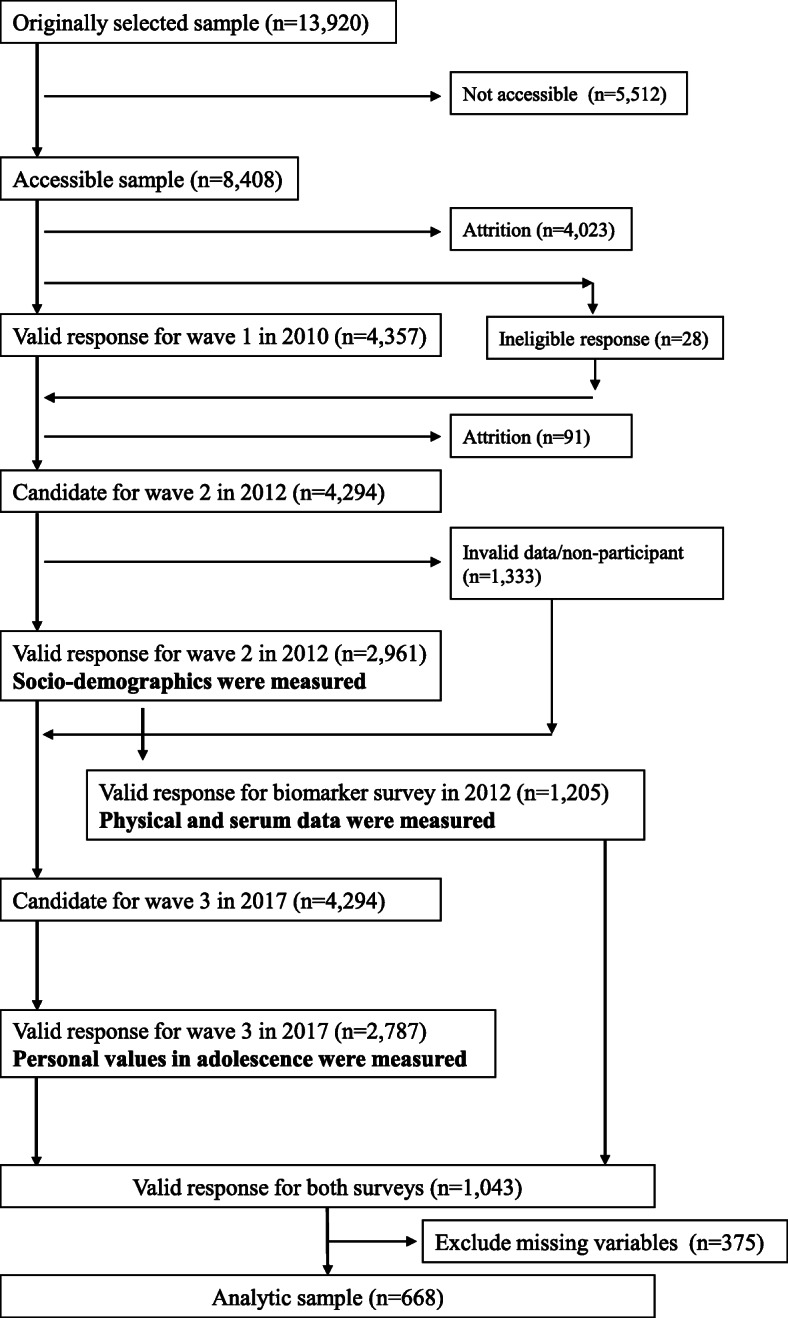
Flowchart of participant recruitment in J-SHINE

J-SHINE data was collected at three points: between July 2010 and February 2011 (wave 1), July and December 2012 (wave 2), and October 2017 (wave 3). Target participants were randomly extracted from the Basic Resident Register of adults aged 25–50 years old from four municipalities in Japan (two in the Tokyo metropolitan area and two in neighboring prefectures). Wave 1 sample sizes were 4357 (valid response rate: 31.3% = valid data (*n* = 4357) / originally selected sample (*n* = 13,920); cooperation rate: 51.8% = valid data (*n* = 4357) / accessible sample (*n* = 8408)). Wave 2 captured *n* = 2961, 69.0% of wave 1 participants. Researchers invited all 2961 who answered the wave 2 survey to undergo physical measurement and serum sampling using a self-administered finger-prick blood sampling kit (Demecal Kit; Leisure Inc., Tokyo, Japan). The subpopulation that underwent biomarker measurements was 1205. *N* = 2787 answered the questionnaire in Wave 3 (response rate = 64.9%). We used the data from waves 2 and 3. People who agreed to participate in the study completed a self-administered questionnaire using a computer-aided personal instrument (CAPI). People who were unfamiliar with computers sat for a personal interview according to the CAPI. Eligible participants offered physical data and a serum sample in the wave 2 survey and completed a questionnaire about personal values in adolescence in wave 3. Those who had incomplete data for these variables and confounders (age and education) in 2012 were excluded. The flowchart of participant recruitment is shown in Fig. 1.

### Measurements

#### Exposure -The personal value in adolescence-

Personal values in adolescence was developed based on Schwartz’s theory of 10 basic values [21, 26]. Although a scale for measuring personal values in Schwartz’s theory was already developed, the scale has been used primarily for adults and not adolescents. The terms and constructs should be locally meaningful for adolescents [27]. A research article that used this scale has already been published [24].

Personal values contain two components: value priorities and commitment to the values. Value priorities were measured by 11 items: economically succeeding, improving society, exploring interests, influencing society, actively challenging, cherishing familiar people, graduating from a famous school, and maintaining a stable life. These items are rated on a seven-point Likert scale (1 = Not at all to 7 = Very important) following the question, “When you were 15–16 years old, how important did you think the following values were in your life?” Test–retest reliability was acceptable (0.556 to 0.729) when examined in another dataset in a 1-month interval, except for the value of “Cherishing familiar people (0.372).” Cronbach’s alphas in the same sample ranged from 0.540 to 0.842. These 11 items can be possibly redefined into Schwartz’s four dimensions: *Self-transcendence* (universalism, benevolence); "Improving society" and "Cherishing familiar people". *Conservation* (conformity, tradition, security); "Not bothering others" and "Maintaining a stable life"; *Openness to change* (self-direction, stimulation, hedonism); "Having and keeping a belief", "Exploring what you were interested in" and "Actively challenging"; *Self-enhancement* (hedonism, achievement, power); "Being highly evaluated by others", "Economically succeeding", "Having influence on society" and "Graduating from a famous school".

Commitment to the values was measured by the Personal Values Questionnaire II (PVQ-II) [22]. The Japanese PVQ-II consists of eight items (e.g., How committed are you to living this value?). Each item is rated on a five-point Likert scale. The internal consistency, concurrent, and structural validity have already been confirmed [28]. In this study, we changed the items from the present to the past tense and instructed the participants to answer the items they had considered the most important when they were 15–16 years old. The sum of the PVQ-II scores was used for analysis; higher scores indicate greater commitment to the values. Test–retest reliability in 2 weeks in another dataset was 0.742, and Cronbach’s alpha was 0.851.

#### Outcomes -Biomarkers and physical health index-

Primary outcomes in this study were important risk factors related to MetS and lifestyle-related disease. Venous samples were collected for low-density lipoprotein (LDL) cholesterol (mg/dL), high-density lipoprotein (HDL) cholesterol (mg/dL), and HbA1c (%, NGSP: National Glycohemoglobin Standardization Program).

Body mass index (BMI, kg/m2) was calculated using the formula; weight (in kilograms) divided by height (in centimeters). Waist circumference (cm) was measured by a tape measure at the level of the umbilicus. Auscultatory systolic and diastolic blood pressure (mmHg) were assessed with a mercury column before and one minute after standing. We took three measurements and used the average.

#### Sociodemographic characteristics

Sociodemographic variables included age and educational status (less than junior high school diploma, high school diploma, some college, more than university degree). These two covariates were used for adjustment in the statistical analyses (partial correlation analysis and multiple linear regression analysis).

### Statistical analysis

First, partial correlation coefficients between the personal values in adolescence and the health outcomes in adulthood were calculated, adjusting for age, educational status, marital status, working status, household income, and all other variables of personal values and biomarkers related to MetS. Second, to examine overall associations between personal values and biomarkers of MetS, a standardized score of a proxy of MetS (Z-score) was calculated by summing the standardized scores of each Mets-related marker, higher scores indicating adverse conditions. We used the Z-score as a dependent variable in the multiple linear regression analysis. Statistical significance was defined as *p* < 0.05. All of the statistical analyses were performed using SPSS 26.0, Japanese version.

### Post-hoc statistical power calculation

After conducting the multiple regression analysis, we calculated the post-hoc statistical power (1-beta) for the significant effects. Among men, the smallest and most significant effect size was a 0.015 increase of R2. As a result, the estimated post-hoc power (1-beta) was 0.51 when the total sample size was 261, if the effect size f2 was 0.015, assuming that the alpha was less than 0.05 (two-tailed), using the G*Power 3 program [29, 30].

## Results

The number of participants with no missing data on the variables used for analysis who were eligible for this study was 668 (261 men and 407 women). The average age of the men was 39.27 (SD = 6.8) years old and that of the women was 38.41 (SD = 6.6). The total population (*n* = 668) was estimated to represent 15.3% of all participants in the J-SHINE cohort. Table 1 shows the descriptive characteristics of this study sample. The frequency distribution of variables showed an almost normal distribution. Compared to those who took the biomarker survey but were dropped from the analysis because of missing variables or not responding to wave 3 (*n* = 537), the final respondents (*n* = 668) were significantly older (mean age, 36.37 and 38,74, respectively, *p* < 0.001), more likely to be married (*p* < 0.001), and had higher household income (*p* = 0.020). There was no significant difference in sex, education status, work status, household income, BMI, waist circumference, SBP or DBP, serum LDL, HDL, or HbA1c between the two samples (data available upon request).

**Table 1:** Demographic and clinical characteristics

	Men (*N* = 261)		Women (*N* = 407)	
	N (%)	Mean (SD)	N (%)	Mean (SD)
Age	261 (100)	39.27 (6.8)	407 (100)	38.41 (6.6)
Education	261 (100)		407 (100)	
Less than junior high school diploma	10 (3.8)		8 (2.0)	
High school diploma	65 (24.9)		90 (22.1)	
Some college	57 (21.8)		190 (46.7)	
More than university degree	129 (49.4)		119 (29.2)	
Marital status	261 (100)		407 (100)	
Married	217 (83.1)		338 (83.0)	
Unmarried	44 (16.9)		69 (17.0)	
Work status	261 (100)		407 (100)	
Employed	254 (97.3)		170 (66.3)	
Unemployed	7 (2.7)		137 (33.7)	
Household income (million Japanese yen/year)	261 (100)		407 (100)	
< 100	4 (1.5)		4 (1.0)	
100–200	3 (1.1)		13 (3.2)	
200–500	67 (25.7)		90 (22.1)	
500–1000	131 (50.2)		212 (52.1)	
> 1000	56 (21.5)		88 (21.6)	
Personal value priority	261 (100)		407 (100)	
Not bothering others		5.45 (1.5)		5.83 (1.2)
Being highly evaluated by others		4.70 (1.5)		5.08 (1.3)
Having and keeping a belief		4.66 (1.5)		4.82 (1.3)
Economically succeeding		4.45 (1.6)		4.05 (1.5)
Improving society		3.71 (1.5)		3.63 (1.3)
Exploring what you were interested in		5.33 (1.4)		5.00 (1.4)
Having influence on society		3.36 (1.4)		2.93 (1.3)
Actively challenging		4.42 (1.4)		4.41 (1.5)
Cherishing familiar people		5.18 (1.4)		5.70 (1.2)
Graduating from a famous school		4.08 (1.7)		4.31 (1.6)
Maintaining a stable life		4.78 (1.5)		4.94 (1.4)
Commitment to Values (PVQ-II^z^)		26.75 (5.1)		26.81 (4.8)
Serum biomarker	261(100)		407 (100)	
LDL^a^(mg/dL)		109.34 (29.0)		101.63 (30.9)
HDL^b^(mg/dL)		59.70 (15.6)		75.36 (17.7)
HbA1c^c^(%,NGSP)		5.50 (0.6)		5.37 (0.3)
Body Mass Index(kg/m^2^)		23.58 (3.2)		21.34 (3.1)
Waist circumference (cm)		87.34 (8.8)		77.94 (9.0)
Systolic blood pressure(mmHg)		125.54 (16.9)		112.37 (14.5)
Diastolic blood pressure(mmHg)		80.28 (13.3)		70.07 (10.7)

^a^ *LDL* low-density lipoprotein

^b^ *HDL* high-density lipoprotein

^c^ *HbA1c* hemoglobin A1C

z *PVQ-II* Personal Values Questionnaire II

Table 2 shows the results of the partial correlation analysis between personal values and biological markers related to MetS. For men, the personal value priority “Having influence on society” was associated with high HDL cholesterol (0.133, *p* = 0.032), and “Cherishing familiar people” with low waist circumference (*r* = -0.129, *p* = 0.049), low SBP, and high DBP (*r* = -0.135, *p* = 0.039; *r* = 0.134, *p* = 0.041). for women, “Not bothering others” was associated with high SBP and low DBP (*r* = 0.125, *p* = 0.015; *r* = -0.123, *p* = 0.017). No significant associations were found after applying Bonferroni corrections.

**Table 2:** Partial correlation(s) for serum outcome in relation to the personal value variables of Japanese adolescents, adjusted by all other variables

	LDL ^a^	HDL ^b^	HbA1c ^c^	BMI ^d^	Waist ^e^	SBP ^f^	DBP ^g^
**MEN** (*N* = 261)							
Not bothering others	-0.041	0.060	0.000	-0.037	0.045	0.062	-0.068
Being highly evaluated by others	-0.031	-0.003	-0.059	-0.021	0.012	-0.014	-0.042
Having and keeping a belief	-0.059	-0.068	0.046	0.005	-0.050	0.078	-0.093
Economically succeeding	0.020	-0.123	0.087	-0.002	0.007	0.071	-0.069
Improving society	0.101	-0.074	-0.101	-0.025	-0.015	-0.094	0.115
Exploring what you were interested in	0.022	0.094	0.101	0.045	0.058	-0.023	-0.039
Having influence on society	-0.048	0.133*	-0.026	-0.039	0.083	0.046	-0.035
Actively challenging	-0.052	-0.027	0.019	-0.040	-0.016	0.102	-0.065
Cherishing familiar people	0.069	-0.025	-0.020	0.083	-0.129*	-0.135*	0.134*
Graduating from a famous school	-0.052	0.012	-0.045	-0.002	-0.011	-0.064	0.083
Maintaining a stable life	-0.036	0.012	0.054	0.029	0.019	0.011	-0.034
Commitment to Values(PVQ-II^z^)	-0.016	-0.084	-0.036	0.010	-0.006	-0.077	0.098
**WOMEN** (*N* = 407)							
Not bothering others	-0.071	-0.065	0.000	0.019	-0.052	0.125*	-0.123*
Being highly evaluated by others	-0.057	0.017	-0.036	-0.035	0.044	-0.025	0.074
Having and keeping a belief	-0.048	0.050	0.085	-0.016	-0.001	0.058	-0.024
Economically succeeding	-0.019	0.009	0.018	0.018	0.042	-0.051	0.053
Improving society	-0.054	0.005	0.019	0.005	-0.037	0.010	-0.026
Exploring what you were interested in	0.059	0.055	0.081	0.058	-0.050	0.026	-0.030
Having influence on society	0.057	0.031	0.066	-0.061	0.074	-0.054	0.059
Actively challenging	-0.024	-0.088	-0.067	-0.017	0.023	0.040	-0.069
Cherishing familiar people	-0.029	0.097	0.031	0.029	0.019	-0.057	0.043
Graduating from a famous school	-0.020	0.063	0.051	-0.031	0.017	-0.025	0.007
Maintaining a stable life	0.016	0.031	0.054	-0.027	-0.010	0.031	0.012
Commitment to Values(PVQ-II^z^)	0.088	-0.008	-0.044	0.029	-0.022	-0.055	0.036

Each coefficient was controlled by age, educational status, marital status, working status, household income and all other variables of personal values and MetS-related markers

^a^ *LDL* low-density lipoprotein, ^b^ *HDL* high-density lipoprotein, ^c^ *HbA1c* hemoglobin A1C, ^d^ Body Mass Index, ^e^ Waist circumference, ^f^ Systolic blood pressure, ^g^ Diastolic blood pressure, ^z^ *PVQ-II* Personal Values Questionnaire II

^*^ *p*-value < 0.05

Table 3 shows the standardized regression coefficients (β) and their p-values between personal values and the Z-score as a proxy of MetS in the multiple linear regression analysis. For men, the personal value priority "Being evaluated by others" was associated with better outcome (β = -0.151, *p* = 0.033). "Economically succeeding" was associated with worse outcome (β = 0.162, *p* = 0.042). For women, the personal value "Graduating from a famous school" was significantly associated with better outcome in the crude model (β = -0.132, *p* = 0.029) but was not significantly after adjustment (β = -0.066, *p* = 0.291). No significant associations were found after applying Bonferroni corrections.

**Table 3:** Coefficient β and *p*-value for z-score as a proxy of MetS with the sum of outcome in relation to the personal value variables of Japanese adolescents

		Z-score^b^							
		**MEN**				**WOMEN**			
		Crude (*N* = 261)		Adjusted (*N* = 261)^a^		Crude (*N* = 407)		Adjusted (*N* = 407)^a^	
No	Items	β	p	β	p	β	p	β	p
V1	Not bothering others	-0.036	0.652	-0.026	0.749	0.005	0.926	-0.067	0.253
V2	Being highly evaluated by others	-0.121	0.092	-0.151	0.033^*^	-0.008	0.882	0.013	0.815
V3	Having and keeping a belief	-0.061	0.435	-0.060	0.438	0.053	0.392	0.039	0.530
V4	Economically succeeding	0.164	0.042^*^	0.162	0.042^*^	0.082	0.194	0.082	0.185
V5	Improving society	-0.011	0.894	0.013	0.873	-0.095	0.159	-0.086	0.194
V6	Exploring what you were interested in	0.057	0.451	0.078	0.301	0.040	0.522	0.063	0.302
V7	Having influence on society	-0.018	0.826	-0.041	0.606	0.100	0.112	0.058	0.346
V8	Actively challenging	-0.029	0.713	-0.031	0.690	-0.074	0.254	-0.030	0.636
V9	Cherishing familiar people	-0.032	0.682	-0.037	0.633	-0.009	0.873	-0.006	0.907
V10	Graduating from a famous school	-0.010	0.891	-0.060	0.470	-0.132	0.029^*^	-0.066	0.291
V11	Maintaining a stable life	0.029	0.728	0.049	0.546	0.043	0.499	0.035	0.569
	Commitment to Values(PVQ-II^z^)	0.060	0.400	0.061	0.385	-0.028	0.617	-0.010	0.855
	*R*^*2*^	0.048	-	0.135	0.413	0.031	-	0.119	0.393
	*ΔR*^*2*^	-	-	0.087	0.010^*^	-	-	0.088	< 0.001^*^

^*^ *p*-value < 0.05

^z^ *PVQ-II* Personal Values Questionnaire II

^a^ Models were adjusted by age, educational status, marital status, working status, and household income

^b^The standardized score for the proxy of Mets (Z-score) was calculated by summing the standardized scores of each Mets-related marker. A high score indicates high-risk metabolic conditions

## Discussion

Some significant associations were found between the personal value priorities in adolescence and MetS-related biomarkers in adulthood: for men, the personal value priority “Having influence on society” was associated with high HDL cholesterol and “Cherishing familiar people” with low waist circumference, low SBP, and high DBP. For women, “Not bothering others” was associated with high SBP and low DBP. However, these associations regarding blood pressure were inconsistent and very weak, thus it is difficult to address the definite meaning of this finding. As an overall composite indicator of MetS, "Being highly evaluated by others" was significantly associated with a good outcome and "Economically succeeding" was significantly associated with a worse outcome, even after adjusting for all variables. The association was still blurry even when considering the cumulative impact of personal values in the cluster analysis. Personal values in adolescence may therefore not be strongly associated with biomarkers related to MetS.

Even though the association found was weak, some personal values in adolescence were certainly linked with metabolic biomarkers in adulthood in the present study. Possible mechanisms can be through behavioral and/or psychological pathways. One of the pathways may be a value-behavior-biomarker link. Personal values in adolescence could be related to forming health-related behaviors [31] in adolescence that would contribute to the development of MetS [6, 7, 32–34]. The other possible pathway is a value-psychological factor-biomarker link. Previous research has suggested that unhealthy (or maladaptive) values may cause psychological distress as anxiety and depression [20] that could increase a risk factor for MetS [35]. The association between personal values in adolescence and MetS in adulthood should be further investigated considering mediating roles of health-related behaviors and psychological distress. Because health-related behaviors and psychological distress may change over the life course in ways that might weaken the observed association between personal values in adolescence and MetS in early adulthood, future research should incorporate time-variant covariates/mediators in the analysis.

In addition, the characteristics of each value that had a significant association with biomarkers should be noted. “Having influence on society” and “Cherishing familiar people” were associated with good outcome of HDL and waist, respectively, for men. Although the priority of “Cherishing familiar people” in men and “Not bothering others” in women significantly influenced blood pressure outcome, the results were inconsistent. As a cumulative indicator related to the MetS of men, "Being highly evaluated by others" was related to a healthy outcome and "Economically succeeding" was related to an unhealthy outcome. For the women, no value that had a provable association was detected. Possible reasons could be how prevalent the values are among the Japanese female and male populations: prevailing values in Japan might be beneficial for health. As previous research indicated, individuals may experience difficulties if their value hierarchies are incongruent with the prevailing hierarchy in their social environments [36]. Sagiv (2000) pointed out that consistency between peoples’ value priorities and their cultural values is critical for well-being [20]. Adaptive choice for preferable personal values may lead to good health by improving well-being [23]. The results from our study seem to be consistent with these findings. “Having influence on society” and “Cherishing familiar people”, which were associated with a better metabolic outcome for men, might be an acceptable value because values related to self-enhancement and self-transcendence are prevalent among men.

This study has several limitations. First, our questionnaire was not the same as Schwartz’s original [21]. Second, there might be recall bias since this study adopted a retrospective design. Life events from adolescence to the time the survey was conducted could have an influence on the recall of values in adolescence. Third, external validity might be limited because dropout rates were relatively high (wave 2; 31%, wave 3: 35.1%). Fourth, lifestyle and obesity at age 15 and as an adult, information that we did not collect in this study, might be a potential confounding factor. Time-variant covariates/mediators such as the alteration of behavioral and psychological factors were not considered. Fifth, estimated post-hoc power (1-beta) was low (0.51 in men). It might be possible that the association would increase with a larger sample size. Sixth, a sufficient number of serum biomarkers to meet the definition of MetS, such as triglycerides, were not measured in this study. Lastly, there would be a possible effect of personality, which might be created antecedent to value. Some personality traits such as higher neuroticism and lower conscientiousness reportedly have been associated with greater risk of metabolic dysfunction from early life [9]. High extraversion among men and low agreeableness among women are known to be related to obesity [37]. The Big Five Personality traits consistently affect health-impairing behaviors and psychopathology [38, 39]. Though value and personality are consistent, a recent meta-analysis found theoretically predictable relations among them, but the constructs are distinct [40]. This study focused on personal values; however, it cannot be denied that personality traits in both adolescence and adulthood affected the association between value and MetS.

Despite these limitations, we have provided evidence that the presence of personal values in adolescence is an important concept for considering health outcome as an adult. Although the overall association was ambiguous, personal values in adolescence might be related to the risk of MetS. Future research would be needed to overcome the limitations of the current study, such as prospective design, using well-validated measurement scale of personal value, and obtaining comprehensive biomarkers to meet the criteria of diagnosis of MetS. Nevertheless, if the effect of adolescents' personal values on MetS is provided, two important policy/practice implications in public health would be stated; (1) education in childhood can be improved to include knowledge about the relation between personal value and health;(2) clinical counseling based on personal value can be an effective approach for health promotion and prevention of MetS. Further evaluation may reveal the impact of personal values on health outcomes through a behavioral and psychological determination of health habits over the lifetime.

## Conclusions

The study found no consistent, strong pattern for the association between personal value priorities in adolescence and MetS-related biomarkers in Japan. However, culturally favorable values (i.e., cherishing familiar people) might be associated with future properties of biomarker related MetS in adults.

## Recommendations

Our findings warrant further research on the association between personal values and MetS and other related health conditions through the life course. The association should be investigated in culturally diverse settings in future research because the function of personal value may differ by culture.

## Data Availability

The dataset obtained from the J-SHINE cohort studies are not publicly available due to a consensus among researchers. Details of the questionnaire adopted in the J-SHINE surveys are available at http:// park.itc.u-tokyo.ac.jp/dhsb/project.html.

## Abbreviations

MetS: Metabolic syndrome
J-SHINE: The Japanese Study on Stratification, Health, Income, and Neighborhood
BMI: Body mass index
AOR: Adjusted odds ratio
OR: Odds ratio
CAPI: A computer-aided personal instrument
PVQ-II: Personal Values Questionnaire II
LDL: Low-density lipoprotein
HDL: High-density lipoprotein
HbA1c: Hemoglobin A1c

## References

[1] Alberti KZ (1999). Definition, diagnosis and classification of diabetes mellitus and its complications: report of a WHO consultation. Part 1, Diagnosis and classification of diabetes mellitus.

[2] Ranasinghe P, Mathangasinghe Y, Jayawardena R, Hills AP, Misra A (2017). Prevalence and trends of metabolic syndrome among adults in the asia-pacific region: a systematic review. BMC Public Health.

[3] Ju SY, Lee JY, Kim DH (2017). Association of metabolic syndrome and its components with all-cause and cardiovascular mortality in the elderly: A meta-analysis of prospective cohort studies. Medicine.

[4] Bray GA (2004). Medical consequences of obesity. J Clin Endocrinol Metab.

[5] Kazaz I, Angin E, Kabaran S, Iyigun G, Kirmizigil B, Malkoc M (2018). Evaluation of the physical activity level, nutrition quality, and depression in patients with metabolic syndrome: Comparative study. Medicine.

[6] Bassi N, Karagodin I, Wang S, Vassallo P, Priyanath A, Massaro E (2014). Lifestyle modification for metabolic syndrome: a systematic review. Am J Med.

[7] Yu JH, Yun CH, Ahn JH, Suh S, Cho HJ, Lee SK (2015). Evening chronotype is associated with metabolic disorders and body composition in middle-aged adults. J Clin Endocrinol Metab.

[8] Yancura LA, Aldwin CM, Levenson MR, Spiro A (2006). Coping, affect, and the metabolic syndrome in older men: how does coping get under the skin?. J Gerontol Series B, Psychol Sci Soc Sci.

[9] Sutin AR, Stephan Y, Terracciano A (2016). Personality and metabolic dysfunction in young adulthood: a cross-sectional study. J Health Psychol.

[10] Berenson GS, Srnivasan SR, Bogalusa Heart Study G (2005). Cardiovascular risk factors in youth with implications for aging: the Bogalusa Heart Study. Neurobiol Aging.

[11] Araujo Fernandes R, Joao Coelho-e-Silva M, Carlos Spiguel Lima M, Ungari Cayres S, Sanches Codogno J (2015). Possible underestimation by sports medicine of the effects of early physical exercise practice on the prevention of diseases in adulthood. Curr Diabetes Rev.

[12] Werneck A, Lima M, Agostinete R, Silva D, Turi-Lynch B, Codogno J (2018). Association between sports participation in early life and arterial intima-media thickness among adults. Medicina.

[13] Prevention(CDC) CfDCa. About Behavioral Risk Factor Surveillance System ACE Data Available from: https://www.cdc.gov/violenceprevention/acestudy/ace_brfss.html.

[14] Jakubowski KP, Cundiff JM, Matthews KA (2018). Cumulative childhood adversity and adult cardiometabolic disease: a meta-analysis. Health Psychol.

[15] Alciati A, Gesuele F, Casazza G, Foschi D (2013). The relationship between childhood parental loss and metabolic syndrome in obese subjects. Stress Health.

[16] Tamayo T, Christian H, Rathmann W (2010). Impact of early psychosocial factors (childhood socioeconomic factors and adversities) on future risk of type 2 diabetes, metabolic disturbances and obesity: a systematic review. BMC Public Health.

[17] Miller GE, Lachman ME, Chen E, Gruenewald TL, Karlamangla AS, Seeman TE (2011). Pathways to resilience: maternal nurturance as a buffer against the effects of childhood poverty on metabolic syndrome at midlife. Psychol Sci.

[18] Appleton AA, Buka SL, Loucks EB, Rimm EB, Martin LT, Kubzansky LD (2013). A prospective study of positive early-life psychosocial factors and favorable cardiovascular risk in adulthood. Circulation.

[19] McDermott JF, Robillard AB, Char WF, Hsu J, Tseng WS, Ashton GC (1983). Reexamining the concept of adolescence: differences between adolescent boys and girls in the context of their families. Am J Psychiatry.

[20] Sagiv LSS (2000). Value priorities and subjective well-being: direct relations and congruity effects. Eur J Soc Psychol.

[21] Schwartz SH (1992). Universals in the content and structure of values: theoretical advances and empirical tests in 20 countries. Adv Exp Soc Psychol.

[22] JT Blackledge J Ciarroch A Bailey. Personal Values Questionnaire II 2010 Available 355 online at: https://app.box.com/v/pvq#pvq/1/53049286/520163710/1.

[23] Patty Ferssizidis LMA, Kashdan TB, Plummer C, Mishra A, Ciarrochi J (2010). Motivation for and commitment to social values: The roles of age and gender. Motiv Emotion.

[24] Yasuma N, Watanabe K, Matsunaga A, Nishi D, Kawakami N (2019). Personal values in adolescence and suicidality: a cross-sectional study based on a retrospective recall. BMC Psychiatry.

[25] Takada M, Kondo N, Hashimoto H (2014). Japanese study on stratification, health, income, and neighborhood: study protocol and profiles of participants. J Epidemiol.

[26] SH Schwartz “Robustness and fruitfulness of a theory of universals in individual human 425 values,” in Valores e comportamento nas organizações [Values and behavior in organizations], 426 eds. A. Tamayo and J.B. Porto (Petrópolis, Brazil: Vozes), pp. 56–95. 2005.

[27] Mousseau AC, Scott WD, Estes D (2014). Values and depressive symptoms in American Indian youth of the northern plains: Examining the potential moderating roles of outcome expectancies and perceived community values. J Youth Adolesc.

[28] Doi S, Yokomitsu K, Sakano Y (2014). Personal Values Questionnaire II: internal 371 consistency and validity. Japanese J Behav Ther.

[29] Faul F, Erdfelder E, Buchner A, Lang AG (2009). Statistical power analyses using G*Power 3.1: tests for correlation and regression analyses. Behav Res Methods.

[30] Faul F, Erdfelder E, Lang AG, Buchner A (2007). G*Power 3: a flexible statistical power analysis program for the social, behavioral, and biomedical sciences. Behav Res Methods.

[31] Liu H, Yu S, Cottrell L, Lunn S, Deveaux L, Brathwaite NV (2007). Personal values and involvement in problem behaviors among Bahamian early adolescents: a cross-sectional study. BMC Public Health.

[32] Drager LF, Togeiro SM, Polotsky VY, Lorenzi-Filho G (2013). Obstructive sleep apnea: a cardiometabolic risk in obesity and the metabolic syndrome. J Am Coll Cardiol.

[33] Young DR, Hivert MF, Alhassan S, Camhi SM, Ferguson JF, Katzmarzyk PT (2016). Sedentary behavior and cardiovascular morbidity and mortality: a science advisory from the American Heart Association. Circulation.

[34] Via MA, Mechanick JI (2016). Nutrition in Type 2 Diabetes and the Metabolic Syndrome. Med Clin North Am.

[35] Watanabe K, Sakuraya A, Kawakami N, Imamura K, Ando E, Asai Y (2018). Work-related psychosocial factors and metabolic syndrome onset among workers: a systematic review and meta-analysis. Obes Rev.

[36] Triandis HC (1989). Cross-cultural studies of individualism and collectivism. Nebr Symp Motiv.

[37] Wimmelmann CL, Lund R, Flensborg-Madsen T, Christensen U, Osler M, Lykke ME (2018). Associations of Personality with Body Mass Index and Obesity in a Large Late Midlife Community Sample. Obesity facts.

[38] Hengartner MP, Kawohl W, Haker H, Rossler W, Ajdacic-Gross V (2016). Big Five personality traits may inform public health policy and preventive medicine: Evidence from a cross-sectional and a prospective longitudinal epidemiologic study in a Swiss community. J Psychosom Res.

[39] Vainik U, Dube L, Lu J, Fellows LK (2015). Personality and Situation Predictors of Consistent Eating Patterns. PLoS ONE.

[40] Parks-Leduc L, Feldman G, Bardi A (2015). Personality traits and personal values: a meta-analysis. Pers Soc Psychol Rev.

